# Supplementary material for: From sequence to enzyme mechanism using multi-label machine learning
Source: BMC Bioinformatics. 2014 May 19;15:150. doi: 10.1186/1471-2105-15-150 (PMC4229970; doi:10.1186/1471-2105-15-150)
Supplement: Additional file 2 — Java code of ml2db. Additional file ml2db_code.tar.gz contains the Java source code to run the multi-label machine learning experiments and save the results to database. The code’s Javadoc is included. [file 1471-2105-15-150-S2.zip › additional file 2/ml2db/ml2db/doc/allclasses-frame.html]

All Classes


# All Classes

- ActiveLearning
- ActiveLearningDatabaseTest
- ActiveLearningTest
- AlDataSaver
- AlDbCreator
- AlEvaluationTable
- AlExperimenter
- AlExperimenterTest
- AlExperimentsTable
- AllActiveLearningTests
- AllArffTests
- AllDataTests
- AllExternalPredictionsEvaluationTests
- AllGraphVizTests
- AllMulanArffTests
- AllMulanLearningTests
- AllMulanPredictionTests
- AllMulanTests
- AllPreliminaryTests
- AllTests
- AllTrainTestsTests
- AlOutputManager
- AnnotationsReader
- AnnotationsReaderTest
- Arff
- ArffGeneratorForFilterTests
- ArffProps
- ArffPropsFilesTest
- ArffPropsFromFile
- ArffPropsOneTest
- ArffPropsQueriesOneTest
- ArffPropsQueriesTest
- ArffPropsQueriesTwoTest
- ArffPropsTableTest
- ArffPropsTwoTest
- ArffRecordsTable
- ArffTest
- AttFiltArff
- AttFiltArffTest
- AttFiltDataSetGeneratorTest
- AttFilter
- AttFilterTest
- AttributeFactory
- AttributeFactoryTest
- AttributesByFrequencyPicker
- AttributeUtils
- AttSetByFrequencyPicker
- AttSetByFrequencyPickerTest
- BRkNNALTest
- BRkNNFewInstances
- BRkNNVisible
- BRkNNVisible.ExtensionType
- ByFrequencyPicker
- ConfidenceCalculator
- ConfidenceCalculatorTest
- ConfidenceMetric
- ConfidenceMetricTest
- ConfidencesStorage
- CrossEvaluator
- CrossEvaluatorTest
- CrossExperimenter
- CrossInputManager
- CrossOutputManager
- DatabaseTest
- DataOne
- DataSetChecker
- DataSetCheckerTest
- DataSetDbLoader
- DataSetDbLoaderTest
- DataSetGenerator
- DataSetGeneratorTest
- DataSetManager
- DataSetManagerTest
- DataSetWriter
- DataSetWriterTest
- DataTableFourTest
- DataTableOneTest
- DataTableTest
- DataTableThreeTest
- DataTableTwoTest
- DataTwo
- DbManagerForTest
- DbManagers
- DebuggingObjectOutputStream
- DistanceExperimentDetailsTest
- DistancesCalculator
- DistancesCalculatorTest
- DistancesStorage
- DistancesStorageTest
- DistancesStoreTest
- EvaluationMetricsTest
- EvaluationParameters
- EvaluationParametersTest
- EvaluationResultsTable
- EvaluationResultsTableTest
- EvaluatorNoAUC
- ExperimentDetails
- ExperimentDetailsTest
- Experimenter
- ExperimenterTest
- FalseNegatives
- FalsePositives
- Folds
- GlobalDistanceMetric
- GlobalDistanceMetricTest
- GlobalDistanceWithConfidenceMetric
- GlobalDistanceWithConfidenceMetricTest
- GlobalPositionStore
- GraphViz
- GraphVizArff
- GraphVizArffColoured
- GraphVizArffColouredTest
- GraphVizArffTest
- GraphvizInstance
- GraphVizPrinter
- GraphVizPrinterTest
- GraphVizTest
- IdentifiedSparseInstance
- IdInstance
- IdInstanceTest
- IdMultiLabelInstances
- IdMultiLabelInstancesTest
- ImportArff
- ImportMulanArff
- InputAndOutputDbManagersTest
- InstancesFiller
- InstancesFillerTest
- InstanceUtils
- InstAttValueMap
- InstAttValueMapTest
- JaccardDistance
- JaccardDistanceStored
- JaccardDistanceTest
- JaccardEuclideanDistance
- LabelRandomPicker
- LabelsByFreqPicker
- LabelsByFreqPickerTest
- LinearMultiLabelNNSearch
- LinearNNSearchSkipInstanceItself
- LinearNNSearchSkipInstanceItselfTest
- LoadFirstValidArff
- MicroFMeasurePrinter
- MulanArff
- MulanArffInstancesLoader
- MulanArffProperties
- MulanArffRecord
- MulanArffRecordTest
- MulanArffTest
- MulanAttributeFactory
- MulanDataSetDbLoader
- MulanDataSetDbLoaderTest
- MulanDataSetGenerator
- MulanDataSetGeneratorTest
- MulanDataSetManager
- MulanDataSetManagerTest
- MulanDataSetWriterTest
- MulanDbWriterTest
- MulanEmitPredictions
- MulanInstancesFiller
- MulanInstancesFillerTest
- MulanLearner
- MulanLearners
- MulanLearnerTest
- MulanSerializer
- MulanTrainExample
- NearestNeighboursByFreqUtility
- NearestNeighboursByFreqUtilityTest
- Neighbourhood
- NeighbourhoodExperimentDetails
- NeighbourhoodExperimentDetailsSerialised
- NeighbourhoodExperimentDetailsTest
- NeighbourhoodNetwork
- NeighbourhoodNetworkTest
- NeighboursPrinter
- NeighboursPrinterTest
- *Picker*
- PredictionsExperimentTable
- PredictionsPerformanceMeasurer
- PredictionsPerformanceMeasurerTest
- PredictionsRecordTable
- PredictionsSaver
- PredictionsSaverTest
- ProjectParameters
- QueryTable
- QuickPredictor
- RandomAttributePicker
- RandomAttributePickerTest
- RandomAttributeSetPicker
- RandomAttributeSetPickerTest
- RandomInstancePicker
- RandomLabelsPicker
- RandomNeigbourhoodPicker
- RandomPicker
- ResultCheckerTest
- ResultsFormatterTest
- Serializer
- SerializerHelper
- SerializerTest
- Statistics
- StatisticsTest
- StatisticsTest
- StratifiedFolds
- TestProjectParameters
- TrainTestEvaluator
- TrainTestEvaluatorSerialised
- TrainTestEvaluatorTest
- TrainTestExperimenter
- TrainTestExperimenterSerialized
- TrainTestExperimenterTest
- TrainTestFullRun
- TrainTestInputManager
- TrainTestOutputManager
- TruePositives
- UtilityMetric
- UtilityTable
- WekaTest
